# Decoding the molecular mechanism of stypoldione against breast cancer through network pharmacology and experimental validation

**DOI:** 10.1016/j.sjbs.2023.103848

**Published:** 2023-10-21

**Authors:** Hina Qayoom, Bader Alshehri, Burhan Ul Haq, Abdullah Almilaibary, Mustfa Alkhanani, Manzoor Ahmad Mir

**Affiliations:** a Department of Bioresources, School of Biological Sciences, University of Kashmir, Srinagar 190006, India; b Department of Medical Laboratory Sciences, College of Applied Medical Sciences, Majmaah University, Almajmaah 11952, Saudi Arabia; c Department of Family & Community Medicine, Faculty of Medicine, Al Baha University, Albaha 65511, Saudi Arabia; d Department of Biology, College of Science, Hafr Al Batin University of Hafr Al-Batin, 31991, Saudi Arabia

**Keywords:** Breast cancer, UALCAN, VEGF, PI3K-Akt, MAPK, Molecular docking, MD simulation, Stypoldione, Network pharmacology

## Abstract

Breast cancer is the primary factor contributing to female mortality worldwide. The incidence has overtaken lung cancer. It is the most difficult illness due to its heterogeneity and is made up of several subtypes, including Luminal A and B, basal-like, Her-2 overexpressed and TNBC. Amongst different breast carcinoma subtypes, TNBC is the most deadly breast cancer subtype. The hostile nature of TNBC is mainly attributed to its lack of three hormonal receptors and hence lack of targeted therapy. Furthermore, the current diagnostic options like radiotherapy, surgery and chemotherapy render unsuccessful due to recurrence, treatment side effects and drug resistance. The majority of anticancer drugs come from natural sources or is developed from them, making nature a significant source of many medicines. Marine-based constituents such as nucleotides, proteins, peptides, and amides are receiving a lot of interest in the field of cancer treatment due to their bioactive properties. The role of stypoldione in this study as a prospective treatment for breast carcinoma was examined, and we sought to comprehend the molecular means/pathways this chemical employs in breast carcinoma. The most promising possibility for an anti-cancer treatment is stypoldione, a marine chemical produced from the brown alga *Stypopodium zonale*. We investigated stypoldione's mode of action in breast cancer using the network pharmacology method, and we confirmed our research by using a number of computational tools, including UALCAN, cBioportal, TIMER, docking, and simulation. The findings revealed 92 common targets between the chemical and breast cancer target network. Additionally, we found that stypoldione targets a number of unregulated genes in breast cancer, including: ESR1, HSP90AA1, CXCL8, PTGS2, APP, MDM2, JAK2, KDR, LCK, GRM5, MAPK14, KIT, and several signaling pathways such as FOXO signaling pathway, VEGF pathway, calcium signaling pathway, MAPK/ERK pathway and Neuroactive ligand-receptor interaction. The examined medication demonstrated a strong affinity for the major targets, according to a docking analysis. The best hit compound produced a stable protein–ligand pair, as predicted by molecular dynamics simulations. Our results are supported by the fact that when in-vitro assays were done on melanoma using stypoldione compound it was found that its mechanisms of action involved the PI3K/mTOR/Akt and NF-kB pathways. This study was set out to inspect the possible value of stypoldione as a breast cancer cure and to get a deeper understanding of the molecular mechanisms by which this drug acts on breast cancer.

## Highlights

• Breast cancer is the primary reason for female fatalities globally.
• It is the most complicated and heterogeneous illness.
• Available therapies including surgery, radiation, and chemotherapy continue to be unsuccessful.
• We examined Stypoldione, a marine natural substance found in brown algae (Stypopodium zonale)
• Using a network pharmacology method, for the first time we elucidated its anti-cancer potential against breast cancer.

## 1 Introduction

Breast cancer (BC) accounts for about 35 % of latest cases of carcinoma and is the frequent cause of mortality for women after heart disease (Siegal et al., 2014, Siegel et al., 2023). With more than 685,000 fatalities and two million new cases in 2021, breast cancer was identified as the number one killer of women worldwide, accounting for 11.7 % of all carcinoma cases that were diagnosed and 15 % of all cancer fatalities, mostly in developing nations (Mir et al., 2023, Bray et al., 2018, Siegel et al., 2023). Breast carcinoma continues to be a serious malignancy among women, with both an elevated incidence and fatality rate. It is a complicated condition that may be broken down into several subgroups depending on the subsistence of hormonal receptors (ER and PR), an increase in the degree of augmentation of the HER2, and other traits. These receptors are missing in TNBC, the most deadly and complicated kind of breast cancer (Anjum et al., 2017). Breast carcinoma continues to be the top priority in biomedical research despite the fact that there has been a lot of progress in the field (Mir et al., 2023, Anastasiadi et al., 2017, Mir and Qayoom, 2023). Therapeutic therapy is still challenging even if the number of fatalities attributable to breast cancer has decreased recently, particularly with regard to metastatic type (Lai et al., 2010, Sofi et al., 2022). For breast cancer diagnosis, the US FDA has authorized a number of medications. But these therapies are extremely costly and have a lot of adverse effects. Some of the typical side effects reported by patients include headaches, lymphedema, musculoskeletal symptoms, fatigue, blood clots, infertility, memory loss, etc. (Waks and Winer, 2019). By identifying novel drug moieties, there are opportunities to lower the number of cancer-related deaths. There are a number of restrictions attached to it, though. The process of developing a brand-new breast cancer treatment involves a number of steps, similar to those used to treat other cancers, including drug design, characterization, production, efficacy and safety testing, and regulatory clearance. The entire process is quite time-consuming and expensive (Gupta et al., 2013, Prasad et al., 2016, Mir et al., 2023a, Mir et al., 2023b). Due to their potential anticancer properties, decreased toxicity, and fewer side effects, herbal medications have received a lot of interest (Ohnishi and Takeda, 2015, Poornima et al., 2016). Herbal medicines are multi-component treatments that target a variety of disease-related genes, proteins, and pathways with numerous chemical substances to produce their desired pharmacological effects (Ohnishi and Takeda, 2015, Poornima et al., 2016). Microalgae, flowering plants (mangroves and other halophytes), macroalgae (seaweeds) and bacteria, actinobacteria, cyanobacteria, and fungus are all parts of the marine flora. The ocean, which makes up over 71 percent of the world's surface, is home to a diverse array of life, with microalgae and microflora accounting for above 90 % of all marine biota (Kathiresan and Duraisamy, 2005). This enormous marine bioresource will provide a lot of opportunity for the development of novel medicines. It is becoming increasingly obvious that the ocean has a wide variety of mineral resources and unique chemical compounds with varied pharmacological activities that might be exploited to create possible pharmaceuticals with higher efficacy and specificity for treating human disorders (Haefner, 2003). Because of their three and half billion years on earth and proficiency in biological synthesis, the marine microorganisms are still nature's finest source of chemicals. To endure the tremendous fluctuations in pressure, salinity, temperature, and other factors that are present in their environment, marine species develop new compounds. These chemicals are distinctive in their variety, structural makeup, and functional characteristics (Mir et al., 2023, Elnahas et al., 2021). Some marine organisms have been shown to be powerful drug sources. These mainly consist of invertebrates like sponges, sea fans, nudibranchs, soft corals, bryozoans, sea hares and tunicates. The creation of therapeutic chemicals is now thought to be a function of the microbial floras found in invertebrates. Floral species are frequently disregarded, with the majority of the search focused on marine faunal species. Although little is known about them, several chemicals originating from marine species have antioxidant and anticancer characteristics (Sithranga Boopathy and Kathiresan, 2010). Stypoldione is a brown alga (*Stypopodium zonale*) derived marine natural substance with an antiquinone functional group is a recently found compound and is quantified to have anti-cancer effect against carcinomas such as Skin (Penicooke et al., 2013, Walter et al., 2022), P388 lymphocytic leukemia cells (White and Jacobs, 1983). In addition, a significant variety of biological and metabolic processes are known to be inhibited by stypoldione. For instance, at high doses, it is known to prevent the formation of spindles, taking up of amino acids, and the incorporation of protein and DNA synthesis, although it is known to restrict fertilized sea urchin egg division at low quantities without impacting spindle organization or chromosomal movement (White and Jacobs, 1983). Additionally, Bovine brain tubulin's *in-vitro* polymerization into microtubules has been demonstrated to be inhibited by stypoldione (O'Brien et al. 1989). Therefore, owing to the effectiveness of stypoldione in multiple melanoma malignancy as has been proved by the *in-vitro* efficacy of the compound performed the group in Brazil (Walter et al., 2022) we explored the pathways taken by Stypoldione against breast tumors utilizing network pharmacology technique followed by numerous computational approaches due to uniqueness of not being researched in breast cancer. Since in today’s scenario breast cancer has been on a significant rise among all carcinoma’s there is a need to find critical and novel therapeutics to which stypoldione seems to be a novel yet effective strategy as validated by our study using several computational tools and network pharmacology approach.

The computational tools such as UALCAN, TIMER, cBioportal etc we utilized to confirm and validate our results further to reach to a conclusive target. A collaborative research strategy called network pharmacology can be utilised to identify complicated disease causes and develop effective treatment plans from a systems viewpoint (Zhang et al., 2013, Hao and Xiao, 2014, Lee et al., 2019, Lee et al., 2020). The multidisciplinary approach combines several scientific disciplines, including computer science, network biology, systems biology, medicine, and pharmacology. To better understand various multicompound, multitarget, multipathway, multipharmacological features of herbal medicine, it has been revealed that network pharmacology is a useful technique. It is therefore widely employed to research the active ingredient(s) and ingredients of herbal medicines as well as the therapeutic targets that are accountable for their pharmacological effects. Network pharmacology studies the relationships among numerous important targets and components that control related systemic processes (Zhang et al., 2013, Hao and Xiao, 2014, Lee et al., 2019, Lee et al., 2020, Qayoom et al., 2022).

## 2 Materials and methodology

### 2.1 Compound: Stypoldione

Information regarding the substance “Stypoldione“ was found in the literature (White and Jacobs, 1983, O'Brien et al., 1989, Sithazard Ratioanga Boopathy and Kathiresan, 2010, Penicooke et al., 2013, Walter et al., 2022). The potential drug-likeness of the potential active ingredient was further investigated. The compound was subsequently put through Swiss ADME analysis (https://www.swissadme.chwebsite). This analysis makes it possible to calculate physical–chemical descriptors and predict the pharmacokinetic properties, drug-likeliness, and pharmacologic chemistry of one or more small molecules, which simplifies drug development in terms of ADMET analysis (Clark and Grootenhuis, 2002, Van De Waterbeemd and Gifford, 2003, Daina et al., 2017).

### 2.2 Identifying a possible gene target for both disease and stypoldione

The target genes for the compound were identified using the Integrative Pharmacology-based Research Platform of Breast Carcinoma and SWISSPROT-TARGET-PREDICTION (https://www.swisstargetprediction.ch/). The Swiss target prediction approach assesses some of a small molecule's most likely macromolecular targets and provides details on chemical compounds and their biological effects (Daina et al., 2019). The structural resemblance to a recognized element was utilised to determine the target. Consecutively to acquire relevant targets pertaining to the chemical of interest, an algorithm was created to choose targets from the Swiss target prediction database using a probability score threshold of less than 0.1. The compilation of genes linked to breast cancer was generated using information sourced from the accessible and comprehensive Gene Cards database (https://www.genecards.org). This database gives inclusive and easily understandable information on both known and predicted genes that are of critical importance in human diseases (Stelzer et al., 2016). The database was examined for genes/proteins with a GIFS (gene cards inferred functionality score) of below thirty by using the keyword “breast tumors,“ and search words such as “triple negative breast cancer” were utilized.

### 2.3 Screening of the disease and the compound's shared target genes

The TCGA (https://www.t2diacod.igib.res.in/), OMIM (https://www.omim.org/), and Gene Atlas of Breast Cancer (https://t2diacod.igib.res.in/) databases were employed to gather data concerning the mutual target genes associated with the disease and the compound (Stypoldione), which were visualized in a Venn diagram. Initially, data from the TCGA database were acquired for breast tumor samples exhibiting the peak 10 % of both over-expressed and under-expressed mRNA genes. Subsequently, potential targets of Stypoldione against genes implicated in breast cancer were identified and represented in a Venn diagram using information from the TCGA database.

### 2.4 Constructing a mutually beneficial network for breast cancer and Stypoldione

By investigating the interactions between proteins, we can pinpoint essential regulatory genes. The STRING database (https://stringdb.org) provides information on verified and anticipated protein interactions across different species, allowing us to access data on these protein–protein interactions (PPI) (Szklarczyk et al., 2017). We employed STRING to identify protein–protein interactions (PPIs), setting a minimum confidence threshold of >0.4. For this experiment, only the species “Homo sapiens“ was considered, and we filtered the confirmed targets with significant ratings > 0.7 before submitting them to STRING. In the end, we acquired PPI data and concentrated our attention on the top 30 proteins with the most significant connections as potential targets for the drug “Stypoldione.”

### 2.5 Kyoto Encyclopedia of genes and genomes with gene ontology pathway enrichment investigation

We employed Shiny GO to perform pathway enrichment and Gene Ontology (GO) analysis on shared target genes within the “Homo sapiens“ species, utilizing data obtained from the Kyoto Encyclopedia of Genes and Genomes (KEGG). The outcomes were visually depicted using bubble charts and bar graphs. Our study encompassed the enhancement of Gene Ontology terms associated with cellular components (CC), biological processes (BP), and molecular functions (MF). Additionally, we utilized bubble and bar graphs to showcase the results of the KEGG pathway enrichment analysis.

### 2.6 Analysis of gene-pathway interactions between disease target and stypoldione

Cytoscape 3.8.0 (available at https://cytoscape.org/, version 3.8.0) was employed to construct an intricate network comprising interactions among a drug, a cancer-related target gene, and a gene pathway. The utilization of this freely available software, Cytoscape, facilitates the visualization of biological processes and molecular interaction networks (Zhang et al., 2021). Furthermore, these connections can be related to genetic expression levels, annotations, and other intricate data, following which Cytoscape can be employed for a thorough examination of the outcomes. To determine the topological characteristics of each node, three parameters can be used. The number of connections linking each node within the network to other nodes is known as “Degree“ and “Betweenness.” The notion of “Closeness“ measures the extent of interconnection, “Closeness” is a metric that functions inversely with the overall count of shortest paths between two nodes in the network. The term “node“ is used to depict the components within the network, including chemicals, target genes, diseases, and pathways. The term “Edge” represents the relationships between these nodes. After assessing the quality indicators using degree-based criteria, we chose all the network's active components that had degrees greater than the average. In network analysis, a node's degree is a simple and direct measure of its centrality (Que et al., 2021).

### 2.7 Clinical significance and the verification of mRNA expression profile of hub genes

We evaluated the mRNA expression levels of essential genes by cross-referencing data from The Cancer Genome Atlas, which can be accessed through the UALCAN database (https://ualcan.path.uab.edu). We established statistical significance using a P-value threshold of 0.05. To assess the expression of these crucial target proteins in both normal and breast cancer tissues, we relied on data from the Human Protein Atlas (HPA) (https://www.proteinatlas.org/). To determine the overall survival rates (OS) associated with these core targets, we used the TIMER cistrome database (https://timer.cistrome.org) and applied a significance threshold with a P-value of 0.05. We then presented the results visually, including hazard ratios. To investigate the genetic uniqueness and connections among the mRNA expressions of these crucial targets, we utilized the cBioPortal tool (https://www.cbioportal.org). We carried out a total of 2,746 analyses using breast cancer samples. Moreover, we employed the Genomic Identification of Significant Targets in Cancer (GISTIC) tool to examine the genomic characteristics of the 12 key targets.

### 2.8 Active compounds-targets docking

This study's goal was to shed some insight on stypoldione's affinity for the KDR protein. The molecule under investigation underwent docking studies using Autodock version 4.2.6 to systematically assess its binding capacity and evaluate the effectiveness of ligand (Stypoldione) binding. Following the addition of non-polar hydrogens to both the protein molecules and the ligand, we saved them in the pdbqt format. The docking grid box has dimensions of 33 × 51 × 52 Å. The construction of the grid boxes had to be done with specific dimensions and 0.3 spacing. In each of the molecular docking studies, there were 50 solutions, 500 population members, 2,500,000 assessments, a limit of 27 generations, and all other parameters were kept at their default settings. The procedure included re-clustering at clustering thresholds of 0.25, 0.50, and 1 to produce RMSD clustering maps. The aim was to pinpoint the most desirable cluster characterized by the least energy change and the greatest concentration of population.

### 2.9 MDS (molecular dynamics simulation)

In the molecular dynamics simulation experiments involving the KDR + Stypoldione complex, the software Desmond 2020.1 was utilized (Shaw et al., 2010). We modeled this system by utilizing the optimized prospective for liquid simulations2005 (OPLS-2005) force field and a precisely defined solvent model. This solvent model included SPC H_2_O molecules confined within a periodic boundary box measuring 10 Å × 10 Å × 10 Å (Bowers et al. 1983, Jorgensen et al., 1983, Chow et al., 2008, Shivakumar et al., 2010). Sodium ion additions were made to the charge to balance it. To replicate the natural conditions/environment, the system was subjected to sodium chloride solutions at an amount of 0.15 M. The system was initially calibrated by retraining it across the protein ligand complexes using a 10 ns NVT (canonical) ensemble. Following that, we conducted equilibration and reductions over a duration of 12 ns, employing an NPT (isobaric-isothermal) ensemble (Martyna et al. 1994). The NPT (isothermal-isobaric) system was created using the Nose-Hoover chain coupling technique, with simulation conditions configured to maintain a pressure of 1 bar, a temperature of 27 degrees Celsius, and a relaxation time of 1.0 picoseconds. We employed a time step of 2 fs (2 fs). Pressure control was achieved by implementing the Martyna-Tuckerman-Klein chain coupling barostat approach with a relaxation time of 2 picoseconds. For the calculation of electrostatic interactions over long distances, we applied the particle mesh Ewald method, setting the Coulomb interaction radius to 9 units (Martyna et al., 1992, Toukmaji and Board, 1996). In each trajectory, computed with 2 fs intervals, we calculated bonded forces using the RESPA integrator. The KDR + Stypoldione complex underwent a concluding production phase lasting 100 ns. To evaluate the reliability of the molecular dynamics simulations, we concurrently examined several parameters, which encompassed root mean square fluctuation (RMSF), radius of gyration (Rg), hydrogen bond count (H-bonds), and root mean square deviation (RMSD).

### 2.10 Investigation of binding free energy

We used the Molecular mechanics with generalized Born and surface area solvation method to calculate the binding free energies of the ligand protein complex. Binding free energies were calculated using 50 simulation frames, utilizing the variable dielectric generalized born model (VGSB) solvation model and the OPLS5 force field. The calculation of binding free energies in molecular mechanics followed an additive methodology, taking into account various energy components, such as hydrogen bonds, van der Waals forces, hydrophobic interactions, electrostatic interactions, self-interactions, covalent interactions, and solvation effects for both the ligand and the protein. These energies are measured in kilocalories per mole (kcal/mol). The below equation may be used to determine Δ Gbind:

$${\Delta G}_{\mathrm{bind}}={\Delta G}_{\mathrm{MM}}+{\Delta G}_{\mathrm{Solv}}-{\Delta G}_{\mathrm{SA}}$$

where

- “ΔGbind“ stands for the binding free energy, which indicates the change in Gibbs free energy when a ligand, such as a compound or substrate, binds to an enzyme or receptor. Evaluating the strength of this interaction between the ligand and its target molecule enables us to predict the ligand's affinity or efficacy.
- “ΔGMM“ represents the difference in free energy between the solvated complex, which includes a protein compound and solvent, and the combined free energies of the individual protein, ligand, and solvent, when compared to each other.
- “ΔGSolv“ measures the impact of solvation on the ligand's attraction to the receptor, accounting for the interactions and structural alterations in the solvent that arise as a result of the complex formation between the ligand and the receptor.
- “ΔGSA“ assesses the alteration in the surface area of both the receptor and ligand that becomes exposed following their binding.

## 3 Results

Stypoldione was chosen for this investigation from the literature. The stypoldione's properties are listed in Table 1. The molecule was further examined for the existence of drug-like characteristics, including pharmacokinetic traits, ADMET (absorption, distribution, metabolism, excretion, and toxicity) parameters, and drug-like nature. The outcomes in Table 2 demonstrate how the molecule is promising because it complies with all ADMET analysis parameters. The drug was further examined using network pharmacology, molecular docking, and MD modeling.

**Table1:** Information regarding the compound's quantitative analysis.

**Compound Name**	**IUPAC Name**	**PubChem ID**	**Molecular formula**	**Molecular weight**
Stypoldione	(1*S*,2*S*,4*aR*,4*bS*,7*S*,8*aR*,10*aR*)-7-hydroxy-2,4*b*,7′,8,8,10*a*-hexamethylspiro [2,3,4,4*a*,5,6,7,8*a*,9,10-decahydrophenantHAZARD RATIOene-1,2′-3*H*-1-benzofuran]-4′,5′-dione	3,034,636	C_27_H_38_O_4_	426.6

**Table 2:** Results of the ADMET analysis.

**Compound Name**	**Mol. Wt.**	**TPSA (A^2^)**	**GI absorption**	**Lipinski’s rule**	**Ghose rule**	**Veber** **rule**	**Egan rule**	**Muegge rule**	**BBB**	**Solubility**	**ADMET screening**
Stypoldione	426.6	63.60	High	Yes	Yes	Yes	Yes	Yes	No	Moderately Soluble	Yes

## 4 Network pharmacology analysis

### 4.1 Building interaction networks and identifying target genes

In the case of Stypoldione, a grand total of 104 potential target genes were identified, as illustrated in Fig. 1 of Supplementary File 1. Simultaneously, the Gene Cards platform helped repossess 12,063 target genes linked to breast tumors. Between breast tumor and Stypoldione, 92 shared common target genes were discovered. In PPI, there were 92 nodes and 279 edges, according to the common target genes PPI diagram (Fig. 2**(a)**).The top 30 most often occurring target genes were displayed in Fig. 2**(b)**. ESR1, HSP90AA1, CXCL8, PTGS2, APP, MDM2, JAK2, KDR, LCK, GRM5, MAPK14, KIT and several target genes displayed elevated levels of protein interactions, suggesting their potential role as central proteins within the network, as indicated in Supplementary Files 2 and 3. The results suggest that the selected compound, “stypoldione,“ exhibited a high attraction to these proteins, suggesting its potential usefulness as a hopeful therapeutic candidate for breast cancer treatment.

**Fig. 1:**
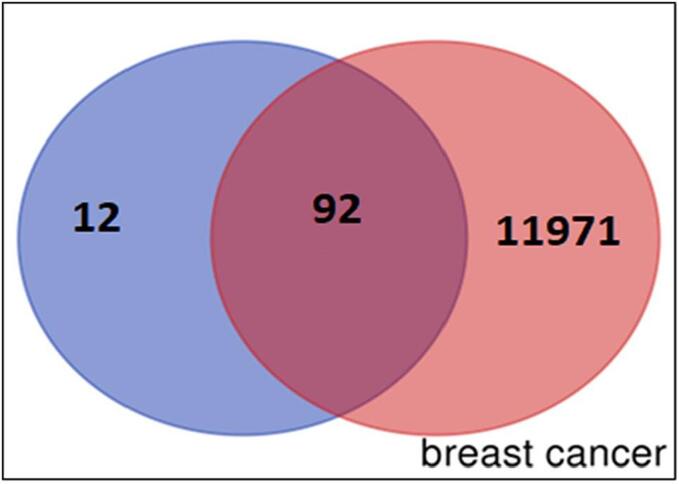
Venn diagram illustrates typical disease and drug target genes. The target genes of stypoldione are shown by the blue circle, the target genes of breast cancer are represented by the red circle, and the common target genes are shown in the coinciding section. The size reflects how many target genes there are.

**Fig. 2:**
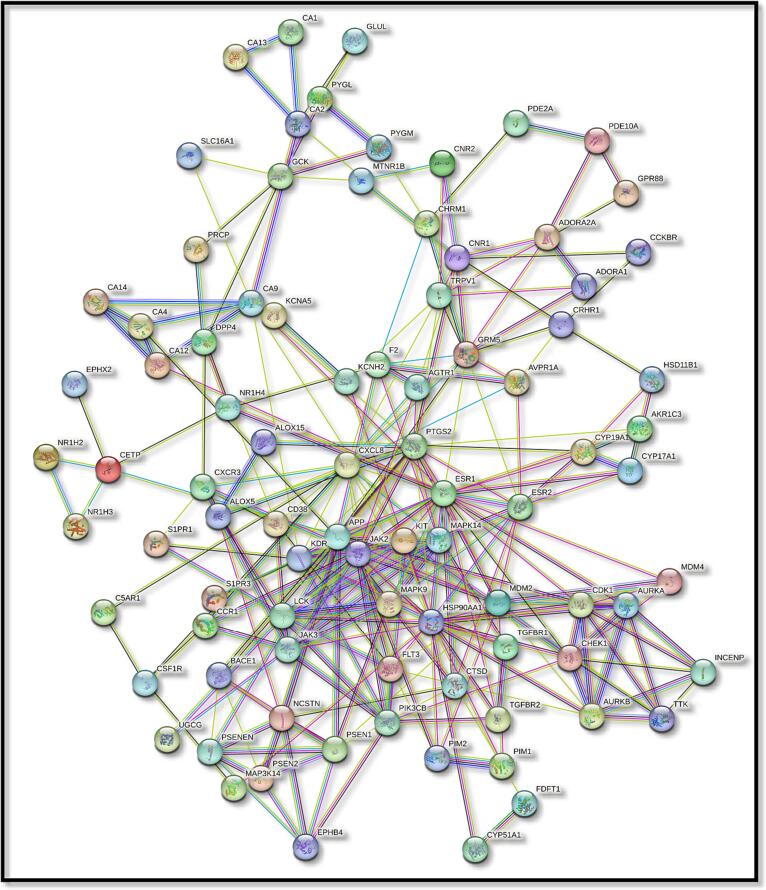

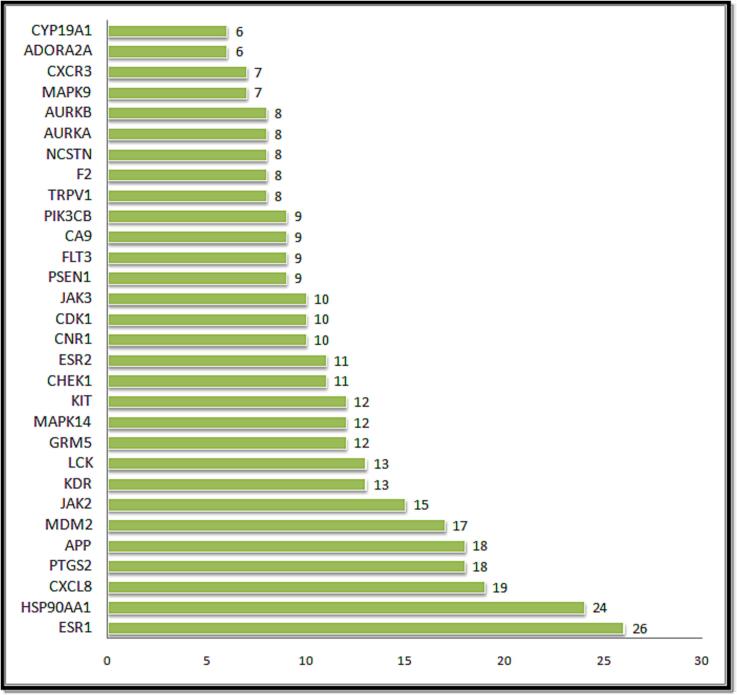
The effect of the interaction between shared target genes' PPI networks is shown in (a). Target genes are represented by nodes, the filled spaces by the 3D structure of the target genes, the edges by associations of target genes, and the colors of the edges by various interactions. For example, the edges are colored cyan and purple for known interactions, green, red, and blue purple for predicted interactions, and chartreuse, black, and light blue for other interactions. (b) The frequency of the 30 most frequent target genes.

### 4.2 Stypoldione key pathways screening for breast cancer treatment

The gene ontology analysis of the mutual target genes, as shown in Fig. 3 of Supplementary Files 4, 5, 6, and 7, indicated that in terms of biological processes, the primary focus was on maintaining homeostasis and regulating transport. On the molecular level, the activity of molecular transducers and signal receptors appeared to be predominant. Additionally, the analysis of cellular components revealed the presence of integral components within the plasma membrane. In Fig. 4 and Table 3 in Supplementary Files 8 and 9, the KEGG pathway analysis of these commonly encountered target genes showcased the top 20 signaling pathways, omitting more general routes. This implies that the use of stypoldione in breast cancer therapy could potentially impact a range of pathways and entail complex interactions within these networks.

**Fig. 3:**
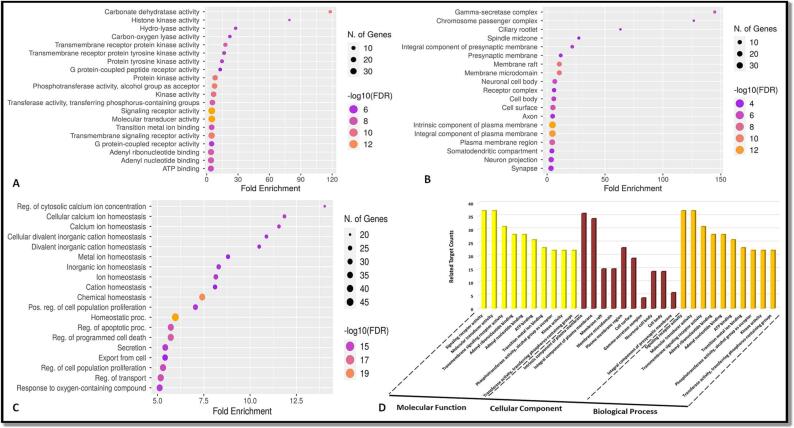
In the top 20 of the GO analysis were molecular function, cellular component, and biological process. A) Bubble diagram showing the Gene Ontology enrichment in the domain of molecular function. B) A bubble diagram displaying the Gene Ontology enrichment study's classification of cellular components. C) A bubble diagram for the Gene Ontology enrichment study of the biological processes category. D) The top processes based on the GO category analysis results and the number of target genes associated with them.

**Fig. 4:**
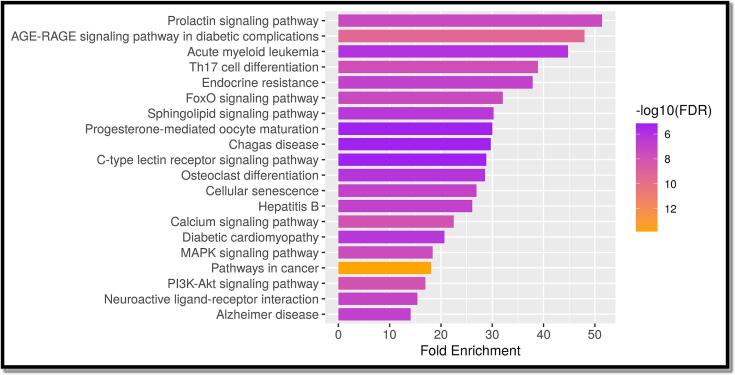

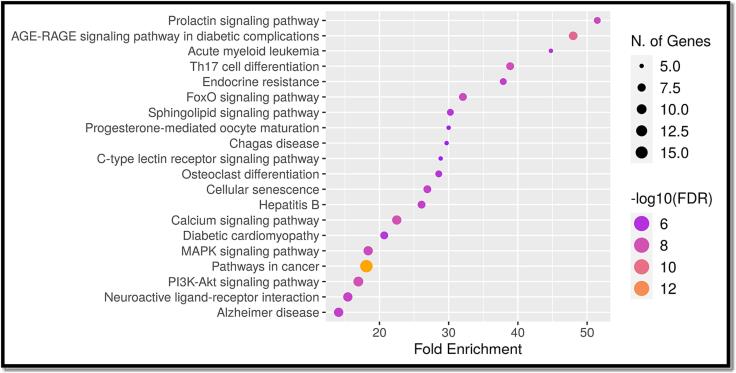
Analysis of the top 20 KEGG pathways (A) Bar plot for pathway enrichment analysis (B) Bubble chart for pathway enrichment analysis.

**Table 3:** Analysis of the Top 20 Pathways.

**Pathway**	**nGenes**	**Fold Enrichment**
Prolactin signaling pathway	6	51.41955
AGE-RAGE signaling pathway in diabetic complications	8	47.99158
Acute myeloid leukemia	5	44.76826
Notch signaling pathway	4	40.67083
VEGF signaling pathway	4	40.67083
Th17 cell differentiation	7	38.88207
Endocrine resistance	6	37.88809
Regulation of lipolysis in adipocytes	3	32.13722
FoxO signaling pathway	7	32.05544
Pancreatic cancer	4	31.57341
CHAZARD RATIOonic myeloid leukemia	4	31.57341
Sphingolipid signaling pathway	6	30.24679
Progesterone-mediated oocyte maturation	5	29.99474
Chagas disease	5	29.69776
C-type lectin receptor signaling pathway	5	28.84109
Osteoclast differentiation	6	28.56642
Colorectal cancer	4	27.90208
Cellular senescence	7	26.91835
Fc epsilon RI signaling pathway	3	26.46594
Hepatitis B	7	26.08238

### 4.3 Network of interactions between a disease target and a prescription active component

**Fig. 5:**
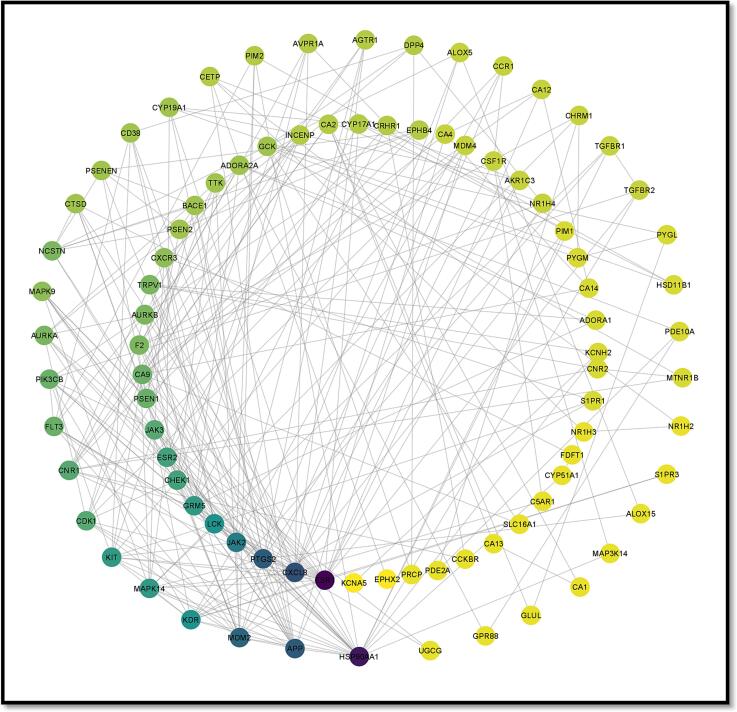

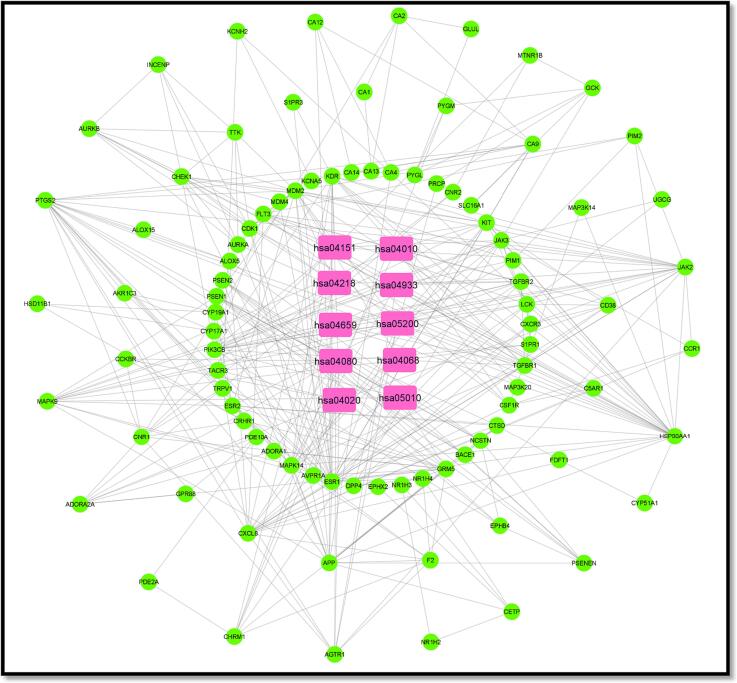

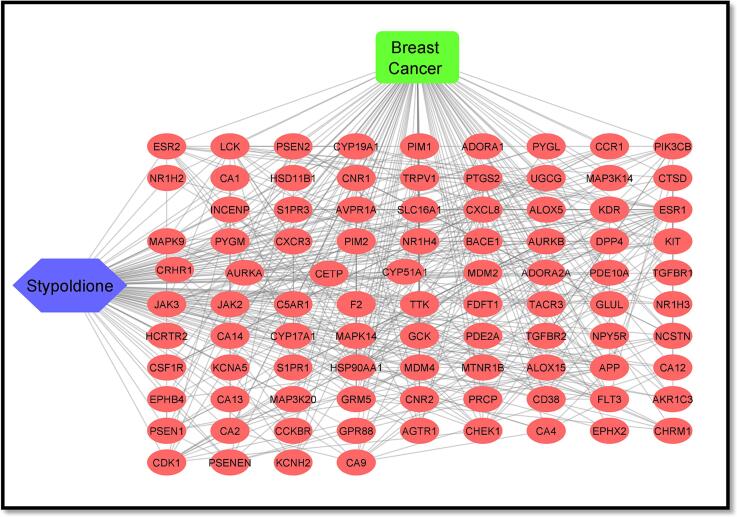
A) Network of protein–protein interactions based on shared targets. B) The major route network for KEGG, as determined by KEGG enrichment analysis. C) Stypoldione (shown by a blue hexagonal node), 92 common targets (represented by a red oval node), etc.

Fig. 5 illustrates the interconnected relationships among the active component, disease, target genes, and pathways. This network encompasses a total of 93 nodes, comprising one active component and 92 target genes. You can also find the interaction network data for this active compound in Table 4. When evaluating the extent of interactions, Stypoldione received a score of 92. These findings suggest that Stypoldione may influence the entire molecular network system rather than solely targeting a single gene. To create the protein–protein interaction network, we utilized protein data from the STRING platform, focusing on common network targets, and applied the Cytoscape tool (as depicted in Fig. 5**A**). The protein nodes are arranged in a hierarchical manner. Additionally, Fig. 5**B** displays the primary pathway network from the KEGG analysis. In Fig. 5**C**, you can observe Stypoldione represented by a blue hexagonal node, along with the 92 shared targets and the connecting edges among them (see Table 5).

**Table 4:** Specifics of the Stypoldione interaction network.

**Component**	**Degree**	**Target gene**
Stypoldione	92	*AURKB, ADORA1, ADORA2A, AGTR1, AKR1C3, ALOX15, ALOX5, APP, AURKA, AVPR1A, BACE1, C5AR1, CA1, CA12, CA13, CA14, CA2, CA4, CA9, CCKBR, CCR1, CD38, CDK1, CETP, CHEK1, CHAZARD RATIOM1, CNR1, CNR2, CRHAZARD RATIO1, CSF1R, CTSD, CXCL8, CXCR3, CYP17A1, CYP19A1, CYP51A1, DPP4, EPHB4, EPHX2, ESR1, ESR2, F2, FDFT1, FLT3, GCK, GLUL, GPR88, GRM5, HCRTR2, HSD11B1, HSP90AA1, INCENP, JAK2, JAK3, KCNA5, KCNH2, KDR, KIT, LCK, MAP3K14, MAP3K20, MAPK14, MAPK9, MDM2, MDM4, MTNR1B, NCSTN, NPY5R, NR1H2, NR1H3, NR1H4, PDE10A, PDE2A, PIK3CB, PIM1, PIM2, PRCP, PSEN1, PSEN2, PSENEN, PTGS2, PYGL, PYGM, S1PR1, S1PR3, SLC16A1, TACR3, TGFBR1, TGFBR2, TRPV1, TTK, UGCG*

**Table 5:** Top 10 signalling pathways based on the ratio of their genes and the genes they interact with.

**Pathway Id**	**Pathway**	**Target genes**
**hsa04151**	PI3K-Akt pathway	PIK3CB HSP90AA1 JAK2 JAK3 FLT3 KDR MDM2 KIT CHAZARD RATIOM1 CSF1R
**hsa04010**	MAPK pathway	MAPK9 MAP3K20 TGFBR1 MAPK14 FLT3 KDR KIT TGFBR2 CSF1R
**hsa04218**	Cellular Senescence	PIK3CB TGFBR1 MAPK14 MDM2 CHEK1 TGFBR2 CDK1
**hsa04933**	AGE-RAGE signaling pathway	MAPK9 PIK3CB JAK2 TGFBR1 MAPK14 PIM1 AGTR1 TGFBR2
**hsa04659**	Th17 cell differentiation	MAPK9 HSP90AA1 JAK2 JAK3 TGFBR1 MAPK14 TGFBR2
**hsa05200**	Pathways in cancer	MAPK9 PIK3CB PTGS2 HSP90AA1 ESR1 JAK2 JAK3 TGFBR1 FLT3 MDM2 PIM1 ESR2 AGTR1 KIT TGFBR2 CSF1R
**hsa04080**	Neuroactive ligand-receptor interaction	CCKBR ADORA2A AGTR1 ADORA1 AVPR1A CHAZARD RATIOM1 GRM5 TACR3 S1PR1
**hsa04068**	FOXO signaling pathway	MAPK9 PIK3CB TGFBR1 MAPK14 MDM2 TGFBR2 S1PR1
**hsa04020**	Calcium signaling pathway	CD38 CCKBR KDR ADORA2A AGTR1 AVPR1A CHAZARD RATIOM1 GRM5 TACR3
**hsa05010**	Alzheimer disease	MAPK9 PIK3CB PTGS2 PSEN1 PSEN2 NCSTN CHAZARD RATIOM1 GRM5 PSENEN

### 4.4 Evaluation of the hub gene's clinical relevance and confirmation of its mRNA expression

**Fig. 6:**
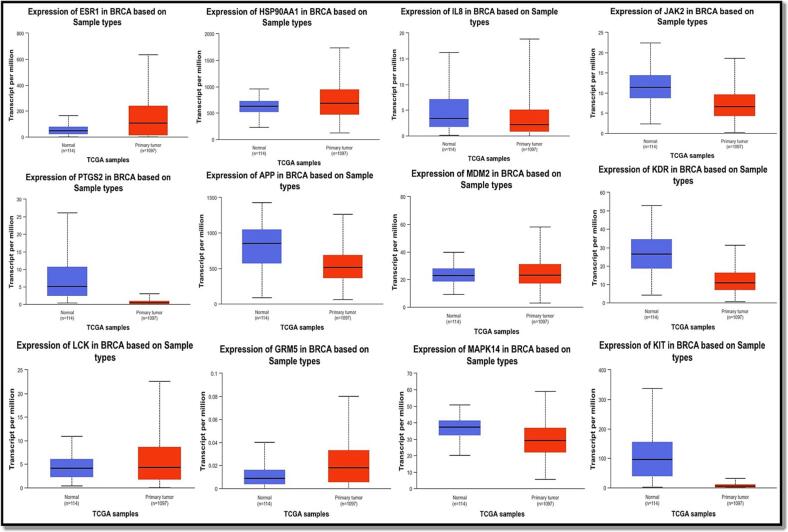

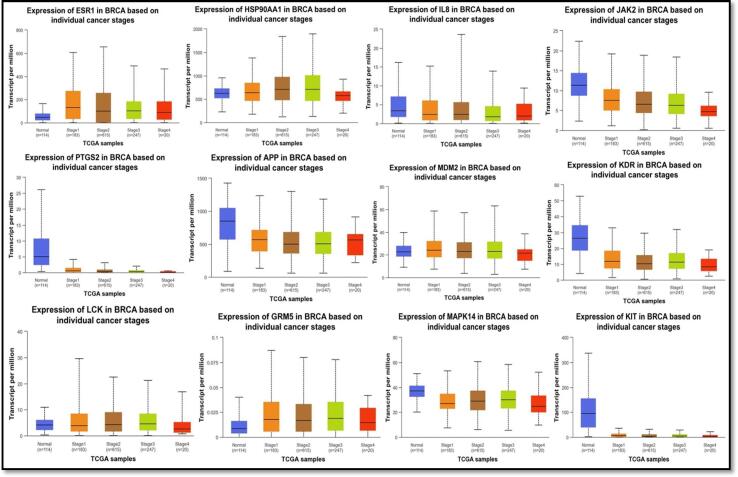
UALCAN mRNA expression in the hub targets is confirmed. (a) Twelve hub targets' mRNA levels in BC tissues and healthy tissues (***P0.001) At various phases of BC tumor growth, the mRNA levels of 12 hub targets are shown in (b) (*P 0.05, **P 0.01, ***P 0.001).

To gain insights into their potential diagnostic and pharmacological significance, we assessed the mRNA and protein expression of 12 central targets selected due to their frequent occurrence in the study. Among patients with invasive breast carcinoma from the TCGA database in UALCAN, it was evident that tumor tissues exhibited significantly elevated levels of ESR1 and GRM5 mRNA, while lower levels of MAPK14 and IL-8 mRNA, both of which are known tumor suppressors, were observed (Fig. 6**a**). In turn, tumor tissues showed significantly lower levels of JAK2, KDR, PTGS2, APP, and KIT mRNA expression, but HSP90AA1, MDM2, or LCK mRNA levels did not decrease noticeably.

Afterward, using the UALCAN database, we inspected the association between 12 hub targets' mRNA levels and different breast cancer tumor phases. ESR1 and GRM5 expression levels were both noticeably higher than average, except that stage four BC tissues did not substantially exhibit increased levels of HSP90AA1, IL-8, APP, or MDM2 as represented in Fig. 6**b**. In contrast, the mRNA levels of PTGS2, LCK, JAK2, KDR, MAPK14 and KIT compared to normal levels, were markedly reduced in 1 to 4 stages of breast cancer tissues.

**Fig. 7:**
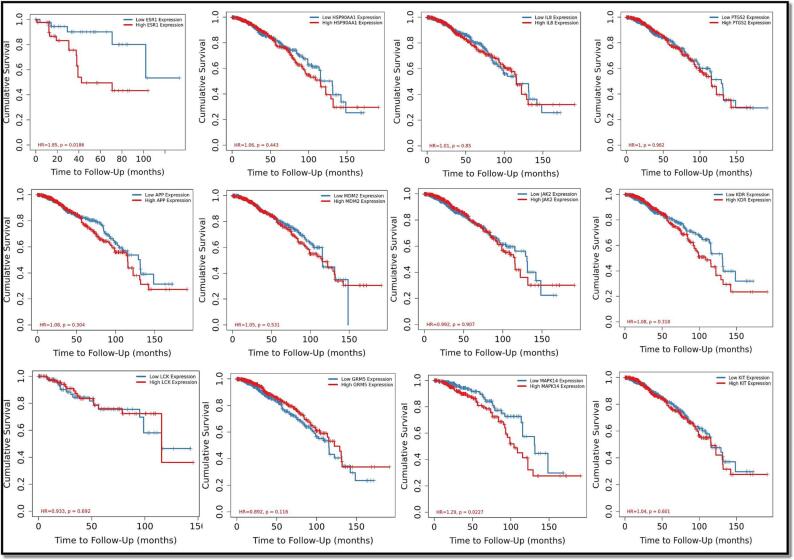
OS analysis of 8 hub targets in Breast Cancer (K-M plot).

We next used the TIMER Cistrome database to examine the prophetic impact of 12 hub targets in breast carcinoma patients. Fig. 7 shows that overall survival was substantially linked with each of the 12 genes such as ESR1 (Hazard Ratio = 1.85, P value = 0.0186), HSP90AA1 (Hazard Ratio = 1.06, P value = 0.443), and CXCL-8 (Hazard Ratio = 1.01, P value = 0.85), PTGS2 (Hazard Ratio = 1, P value = 0.962), APP (Hazard Ratio = 1.08, P value = 0.304), MDM2 (Hazard Ratio = 1.05, P value = 0.531), JAK2 (Hazard Ratio = 0.992, P value = 0.907), KDR (Hazard Ratio = 1.08, P value = 0.318), LCK (Hazard Ratio = 0.933, P value = 0.692), GRM5 (Hazard Ratio = 0.892, P value = 0.116), MAPK14 (Hazard Ratio = 1.29, P value = 0.022) and KIT (Hazard Ratio = 1.04, P value = 0.601). The results demonstrated a substantial relationship between significantly poorer survival and significantly greater expression of KDR, ESR1, MAPK14, APP and HSP90AA1 and low expression of CXCL-8/IL-8, PTGS2, GRM5 and LCK.

**Fig. 8:**
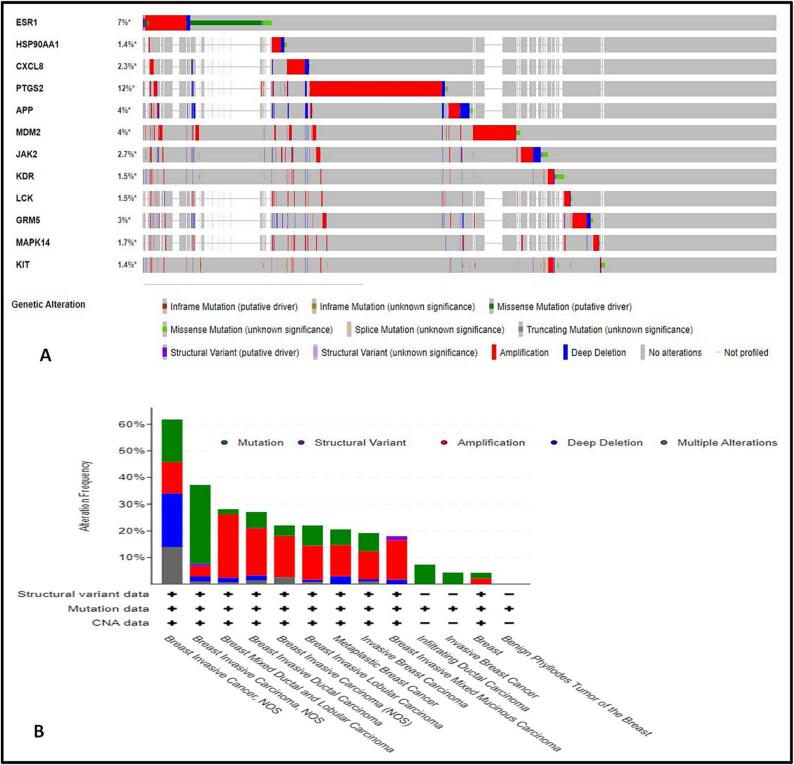
Breast carcinoma patients’ genetic alterations in 12 hub targets (cBioPortal). (A) A visual OncoPrint summary of the genetic alterations discovered in 12 hub targets. (b) A list of the modifications made to 12 hub targets across different breast cancer types.

We proceeded to analyze the incidence and kinds of genetic variations in the 12 central targets among a cohort of 2,746 breast cancer patients using the cBioPortal tool. The individual rates of gene modifications varied, ranging from 1.4 % to 12 %. Notably, PTGS2 exhibited the highest degree of variability at 12 %, while HSP90AA1 and KIT displayed the lowest modification rates at 1.4 %, as indicated in Fig. 8**a**. Collectively, the overall alteration rate for these 12 central targets amounted to 42.5 %. Moreover, when considering diverse breast carcinoma types, the genetic variation rates spanned from 0 % to 100 %, as illustrated in Fig. 8**b**.

**Fig. 9:**
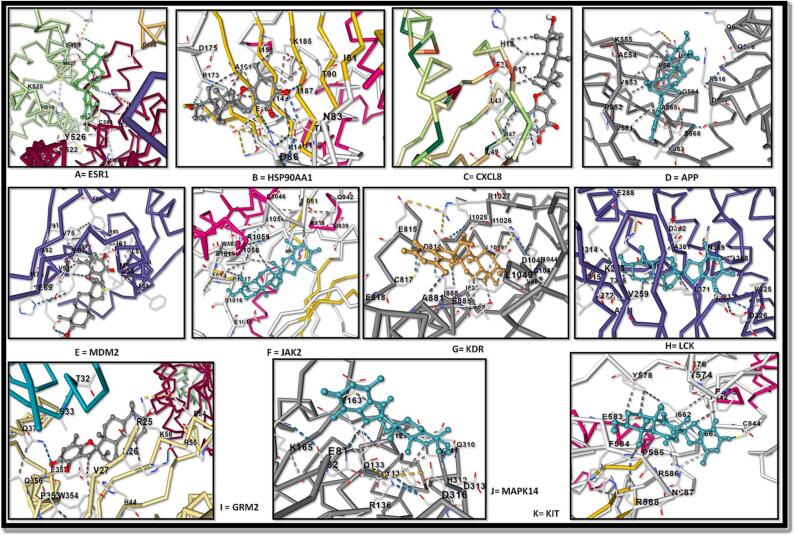
Molecular docking of stypoldione with eleven hub genes: (A) ESR1, (B) HSP90AA1, (C) CXCL8, (D) APP, (E) MDM2, (F) JAK2, (G) KDR, (H) LCK, (I) GRM2, (J) MAPK14, (K) KIT.

This was followed by molecular docking analysis of stypoldione with the respective 11 hub target genes represented in Fig. 9 and Table 6. After which, the best interaction complex was chosen for further analyses.

**Table 6:** Docking results.

**Docking with protein**	**Full Form**	**Dock Score** **(kcal/mol)**
ESR1	Estrogen receptor alpha	−7.9
HSP90AA1	Heat shock protein HSP 90-alpha	−7.7
CXCL8	C-X-C Motif Chemokine Ligand 8	−7.2
APP	Amyloid precursor protein	−8.5
MDM2	Murine double minute 2	−7.8
JAK2	Janus kinase 2	−7.6
KDR	Vascular endothelial growth factor receptor 2	**−9.8**
LCK	Lymphocyte-specific protein tyrosine kinase	−8.7
GRM5	Glutamate Metabotropic Receptor 5	−8.7
MAPK14	Mitogen-Activated Protein Kinase 14	−8.6
KIT	KIT Proto-Oncogene, Receptor Tyrosine Kinase	−8.4

### 4.5 Molecular docking investigations

**Fig. 10:**
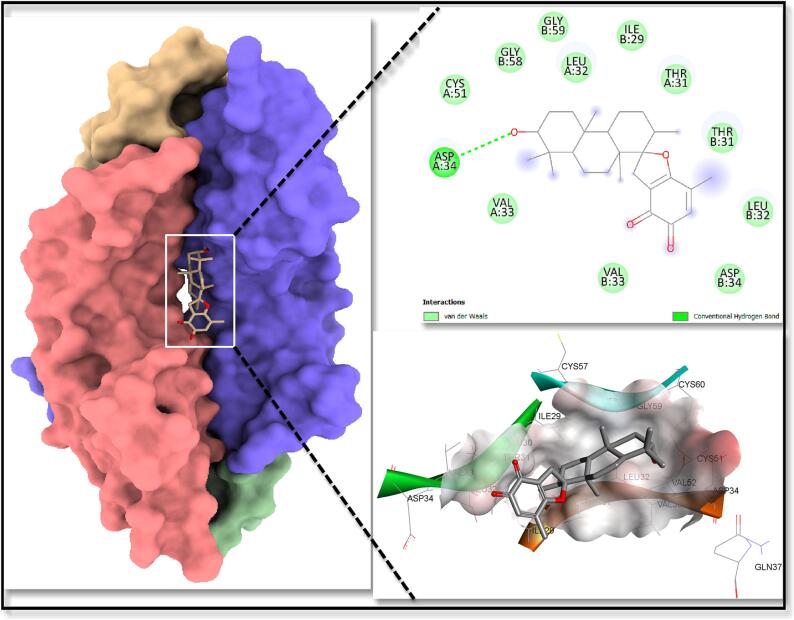
Shows the KDR's molecular surface with Stypoldione attached in a deep hole. The connections between ligand and protein are visible in the right top view of the 2D interaction, whereas the highly charged core ligand-bound site is visible in the right down charged surface.

The binding energy ratings of all clusters were estimated from the best huddle (95 %) that is contained within the tightest RMSD of 0.25. Stypoldione has the highest binding potential for the KDR and the shortest binding affinity (G- 9.8 kcal/mol). Fig. 10 depicts the formation of van der Waal interactions with certain residues during the interaction of the Stypoldione with KDR as opposed to the usual hydrogen bonds that were established with the Asp34 residue. The molecular surface image depicted the interaction between the concave, saddle-shaped core and the ligand Stypoldione, as shown in Fig. 10. At the same time, the image of the charged surface demonstrates the manner in which Stypoldione engages with the electrically charged amino acid residues located within the binding pocket.

### 4.6 Studying molecular dynamics with simulation

**Fig. 11:**
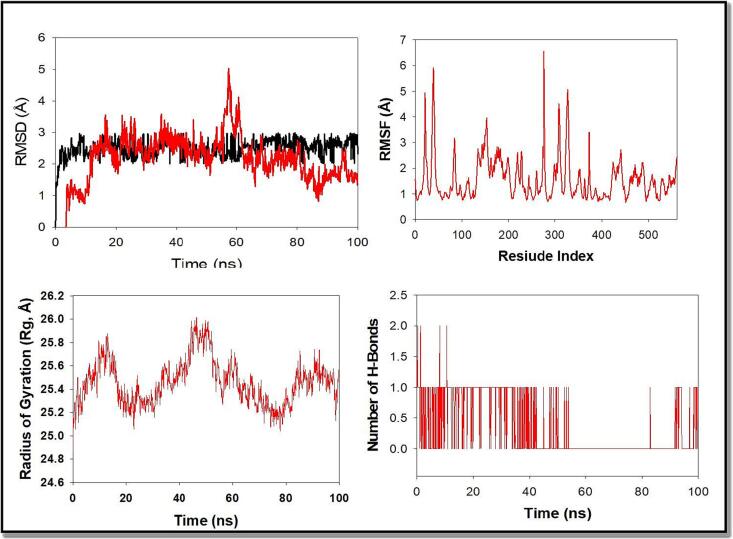
Molecular dynamics simulation study of 100 ns trajectories of (A) C-backbone RMSD of KDR(black) and RMSD of Stypoldione (red), (B) RMSF of C-backbone of KDR, (C) Radius of gyration (Rg) of C-backbone of KDR + Stypoldione, and (D) Formation of hydrogen bonds in KDR + Stypoldione.

To assess the constancy and union of the KDR + Stypoldione complex, we conducted molecular dynamics and simulation (MD) investigations. During a simulation lasting one hundred nanoseconds, we observed consistent conformations, as evidenced by comparing the RMSD values. When KDR was coupled to Stypoldione, the C-backbone showed a displacement of 2.5 in terms of RMSD, while the ligand exhibited an RMSD of 1.8, as shown in Fig. 11**A**. Importantly, all Root mean square deviation values remained within the acceptable range of 3 or below. The consistent RMSD plot throughout the simulation indicated favorable convergence and persistent conformations. Considering the ligand's increased affinity, it suggests that the Stypoldione-KDR complex is highly stable. The RMSF plot of the KDR protein with Stypoldione displayed minor fluctuations in residues 15–25, 60–72, 145–153, and 170–220, which could be associated with greater flexibility, as shown in Fig. 11**B**. The RMSF plots show that the protein retains its flexibility in the conformations when bound to the ligand throughout the simulation. We evaluated the protein's compactness using its Radius of Gyration (Rg). In this study, the KDR C-backbone bound to Stypoldione consistently exhibited an Rg value ranging from 25.2 to 25.4, as illustrated in Fig. 11**C**. The consistently stable Rg value strongly indicates that the protein maintains a condensed structure while interacting with the ligand. Furthermore, the robustness and endurance of the complex were reinforced by the substantial count of hydrogen bonds identified between KDR and Stypoldione throughout the entire one hundred nanoseconds of the simulation, as depicted in Fig. 11**D**.

### 4.7 Calculations using the generalized born surface area (MM-GBSA) technique of molecular mechanics

**Fig. 12:**
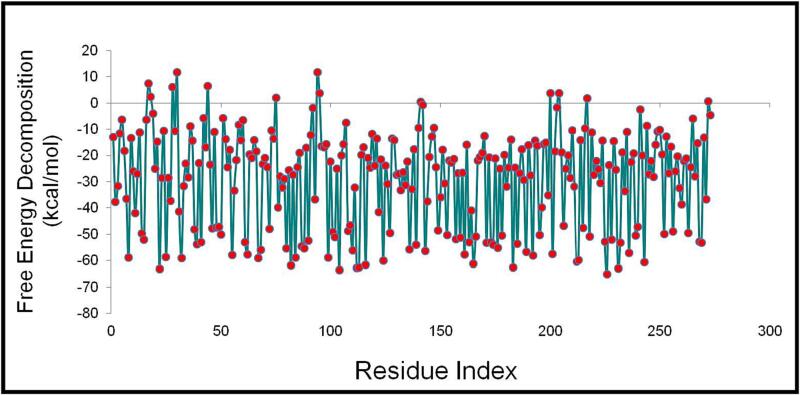
Free energy decomposition of individual residues of protein during ligand binding.

We employed the molecular dynamics simulation trajectory to assess the binding capacity, free energy, and other associated energies in the MM-GBSA format for the KDR + Stypoldione complex. The findings presented in Table 7 and Fig. 12 indicates that ΔGbindCoulomb, ΔGbindvdW, and ΔGbindLipo were the predominant factors contributing to the stability of the simulated complexes, while ΔGbindCovalent and ΔGbindSolvGB were the primary factors leading to their instability. Notably, the combination of KDR + Stypoldione exhibited significantly lower binding free energy, indicating a strong binding affinity. These findings not only support the potential of Stypoldione but also highlight its ability to form stable protein–ligand complexes and effectively interact with the target protein.

**Table 7:** The binding free energy components of KDR + Stypoldione were estimated using MM-GBSA.

**Energies (kcal/mol)**	**KDR + Stypoldione**
**ΔG_bind_**	−56.83 ± 2.0
**ΔG_bind_Lipo**	−20.09 ± 2.0
**ΔG_bind_vdW**	−47.48 ± 2.0
**ΔG_bind_Coulomb**	−3.46 ± 1.0
**ΔG_bind_H_bond_**	−0.007 ± 0.0
**ΔG_bind_SolvGB**	15.28 ± 2.0
**ΔG_bind_Covalent**	1.10 ± 0.5

### 4.8 Calculations using principal component analysis

**Fig. 13:**
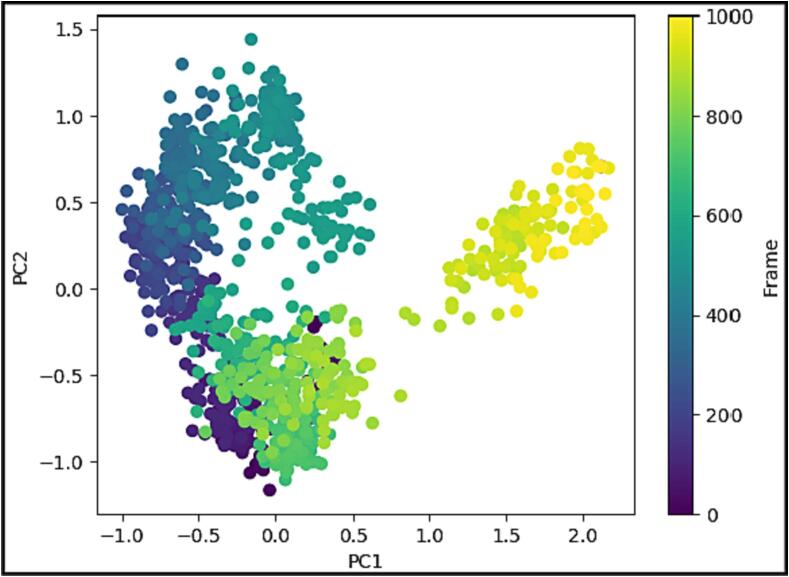
Using the MD trajectory for 100 ns, a PCA analysis on the Eigen vectors of 1000 frames in Cartesian coordinates.

The simulations of the molecular dynamics of the protein–ligand complex's trajectory were put through a PCA, allowing for the examination of the random, global movement of amino acid residue atoms (Fig. 13). This analysis identifies the increased flexibility of the trajectories, which can be attributed to the non-correlated global motion of the protein structure. A covariance matrix was used to track the movement of internal coordinates during a period of 100 ns in a three-dimensional region. Orthogonal sets, specifically eigenvectors, were employed to describe the coherent movement observed in each trajectory. When the drug was coupled to KDR, the C atoms' MD simulation trajectory had a more unorganized orientation in PC1 and PC2 modes, tending towards a negative correlation (Fig. 13). However, the final 100 frames shifted towards the origin, displaying a positive correlation (Fig. 13, yellow). This finding suggests that the protein, when bound to ligand, experiences periodic motion in the molecular dynamics trajectories due to constant conformational global movement.

## 5 Discussion

**Fig. 14:**
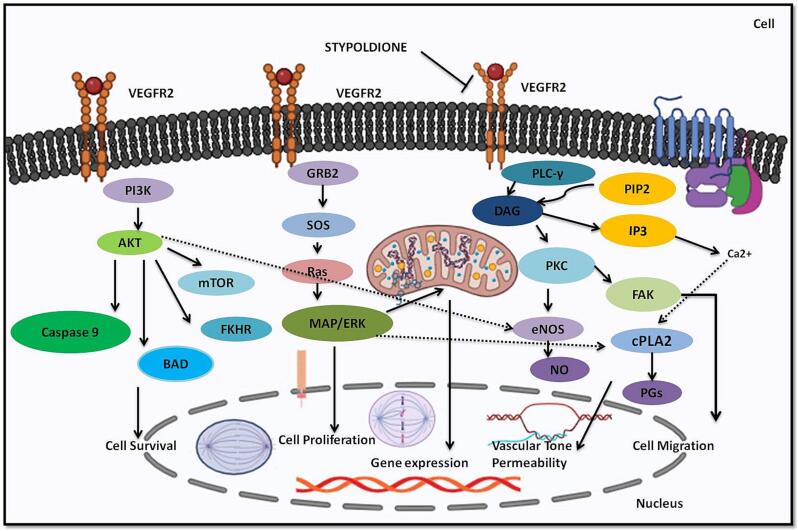
Overview of the involvement of VEGFR2 in various signaling pathway in breast cancer and inhibitory action of Stypoldione on VEGFR2 to reduce the breast cancer metastasis.

**Fig. 15:**
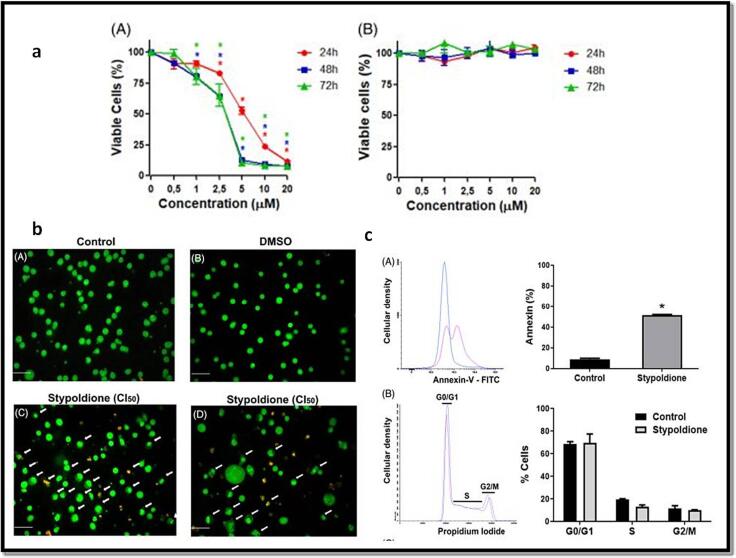
In-vitro assays validated by Brazilian group on stypoldione (a) Results showing effectiveness of stypoldione on multiple myeloma cells by MTT assay (b) Represents apoptosis evaluation in multiple myeloma cells treated with stypoldione (c) Annexin V assay and cell cycle evaluation in multiple myeloma cells after incubation with stypoldione [With permission from Walter LO et al, 2022].

Statistical data reveals that breast carcinoma is the majorly prevalent recurring carcinoma among females and ranks as the fifth leading reason of mortality on a global scale. In the year 2020, over two million women across the world were diagnosed with breast cancer for the first time, constituting 11.7 % of all cancer diagnoses (Mir and Sumaya, 2023, Qayoom et al., 2021, Sung et al., 2021). Surgical procedures and radiation therapy are employed for both diagnostic and therapeutic purposes in the control of breast carcinoma, addressing the malignancy within the breast and lymph nodes. Additionally, treatments like chemotherapy or hormonal therapy are utilized to decrease the risk of cancer development (Mir et al., 2023, Qayoom and Bhat, 2020, Qayoom et al., 2023, Wanchai et al., 2023). Additionally, recent treatment approaches for breast cancer focus on targeting different metabolic pathways, conjugated antibodies, and immunotherapy (Burguin et al., 2021, Qayoom et al., 2021). Regrettably, breast carcinoma and its diagnosis can extensively affect an individual's physical and psychological well-being. Persistent pain, lymphedema, fatigue, and cognitive impairment are some examples of enduring physical alterations that may result from breast cancer treatments (Jan et al., 2023, Bhattacharyya et al., 2020; Mehraj et al., 2021). The majority of the chemical components found in nature is either natural products themselves or are produced from them. The ocean, which makes up about 70 % of the planet, is home to numerous creatures and is therefore a great source of biological substances. Numerous substances with marine origins are useful in pharmaceutical and therapeutic research (Casertano et al., 2020, Mehraj, 2022). Marine chemicals have a variety of functions, including antibacterial, antiviral, antifungal, anti-inflammatory, and antitumor. Various commercial substances with marine origins have shown anticancer properties (Casertano et al., 2020, Carroll et al., 2021). As of October 2020, there were 14 marine-derived chemicals accessible on the market, and 9 of them are utilised as anticancer medications, according to marine pharmacology. Additionally, 19 anticancer substances derived from marine sources were undergoing various stages of clinical testing [Clinical Pipeline Marine Pharmacology [(accessed on 23 March 2021)]; Availableonline: https://www.midwestern.edu/departments/marinepharmacology/clinical-pipeline.xml]. Additionally, many articles have demonstrated the anticancer properties of compounds derived from marine sources *in-vitro* or *in-vivo*. The anticancer substances obtained from marine sources mostly come from cyanobacteria, sponges, tunicate, bacteria, and fungi, as well as mollusks and cyanobacteria (Dyshlovoy et al., 2023). Stypoldione, an o-quinone compound, is derived from the brown algae known as *Stypopodium zonale*. This specific seaweed is classified under the Phaeophyceae class and belongs to the Dictyotaceae family (O'Brien et al. 1986). Research indicates that stypoldione in its pure form has the potential to interfere with the cell cycle of sea urchin embryos and inhibit the formation of microtubules in bovine brain cells (O'Brien et al., 1983, O'Brien et al., 1989, Penicooke et al., 2013). Additionally, stypoldione-containing algae extracts demonstrated anti-tumor activity against human melanoma cells. Stypoldione's isolated impact on cancerous cells has not yet been studied (Rocha et al., 2007). To ascertain and confirm the molecular processes used by stypoldione in breast cancer, we thus carried out an NP analysis. The current study found that stypoldione targets several deregulated genes in breast cancer, including ESR1, HSP90AA1, CXCL8, PTGS2, APP, MDM2, JAK2, KDR, LCK, GRM5, MAPK14, KIT, and signaling pathways such as VEGF pathway, PI3K-Akt pathway, FOXO signaling pathway, MAPK pathway, calcium signaling pathway and Neuroactive ligand-receptor interaction. The expression of the resultant genes was validated using several databases such as TCGA, UALCAN, cBioportal and TIMER. Angiogenesis and vasculogenesis are highly dependent on the signalling proteins referred to as vascular VEGFs. Angiogenesis, which is driven by VEGF, is necessary for the growth and spread of tumors (Carmeliet, 2005). The most significant pro-angiogenic factor at the moment has been identified as VEGF (Mir et al., 2023, Kiselyov et al., 2007, Grothey and Galanis, 2009). Vascular Endothelial Growth Factor receptors (VEGFRs) interact with endothelial cells to activate the MAPK and PI3K/Akt signal pathways, which successively enhance endothelial cell recruitment and proliferation (Carmeliet, 2005, Hicklin and Ellis, 2005, Scott and Mellor, 2009). In humans, the VEGF kinase receptors encompass VEGFR-1, VEGFR-2, and VEGFR-3. Unlike VEGFR-3, which is primarily linked to lymphangiogenesis, VEGFR-1 has a vital function in stimulating the proliferation of monocytes and macrophages, as well as facilitating the release of progenitor cells from the bone marrow Fig. 14 (Zhang et al., 2010, Mir et al., 2020). VEGFR-2, the primary controller of angiogenic signaling triggered by VEGF, governs the development, infiltration, and movement of vascular cells (Lee et al., 2010). VEGFR-2 is comprised of several components, including an internal catalytic domain that possesses tyrosine kinase activity, a transmembrane segment, and an extracellular domain responsible for binding to VEGF. When the immunoglobulin-like regions within the extracellular domain interact with VEGF, they come together in pairs (dimerize). This pairing process triggers autophosphorylation in the intracellular catalytic domain by capturing ATP. The majority of VEGFR-2′s phosphorylation sites are found on tyrosines 1175 and 1214, and these sites activate signaling pathways involving PLC, PI3K, AKT, p38MAPK, and p42/44 MAPK (Yilmaz et al., 2003). In this investigation, we used the programme Auto Dock version 4.2.6 to carry out the molecular docking analysis (Morris et al., 2008) study showed stypoldione's higher affinity for the VEGF protein involved in the VEGF/VEGFR2 (KDR) signalling cascade. The molecular dynamics simulations that produced substantial results were used to further validate the findings. Based on the results of docking scores, the drug demonstrated promising effects on the VEGFR2 (KDR) protein. Stypoldione exhibited superior effectiveness against VEGFR2 compared to all other selected targets. It snugly fits within the binding sites of the VEGF protein and forms favorable interactions with key amino acid residues (see Fig. 10). Notably, Stypoldione displayed the highest binding affinity for KDR, with the lowest binding energy recorded at −**9.8** kcal/mol. It predominantly forms van der Waal interactions with specific residues, as opposed to the typical hydrogen bonds observed with the Asp34 residue. Given Stypoldione's strong affinity for VEGFR2 in these molecular docking studies, it suggests a potential therapeutic application. Furthermore, molecular dynamics simulations were utilized to evaluate the durability of the VEGFR2-Stypoldione compound. The RMSD graphs (see Fig. 11**A and B**) revealed consistent trends throughout the simulation, indicating that Stypoldione effectively maintains its position within the VEGFR2 binding site. The Rg graphs indicated that VEGFR2 remained compact during the simulation, reflecting a stable protein backbone. The examination of alterations in residues throughout the simulation demonstrated a decrease in the exposed surface area of the target following the binding of the ligand. This research underscores Stypoldione's potential as a promising anti-cancer drug for breast cancer treatment, substantiated by a range of molecular mechanisms. It's important to note that these findings are based on a theoretical approach and require further experimental validation. Notably, a Brazilian research team investigated this compound's efficacy against multiple myeloma and identified its mechanisms of action involving the NF-kB and PI3K/mTOR/Akt pathways Fig. 15. This served as the foundation for our study, where we applied a network pharmacology approach to explore Stypoldione's mechanisms of action in breast cancer, an area where it has not been previously studied. Unfortunately, due to the unavailability of this compound for commercial purposes, in-vitro analysis wasnot feasible for our research.

## 6 Conclusion

In our ongoing study, we utilized a network pharmacology approach to examine the molecular mechanism of a recently identified marine compound called Stypoldione, which was extracted from the brown seaweed Stypopodium zonale. Following that, we validated our findings through molecular docking and MD simulation experiments. According to the outcomes of the network pharmacology analysis, Stypoldione exhibits the potential to influence numerous top-ranking signaling pathways, which encompass, among others, the VEGF pathway, PI3K-Akt pathway, FOXO signaling pathway, MAPK pathway, neuroactive ligand-receptor interaction, and calcium signaling pathway. These findings align with prior research that employed molecular docking and computer simulations, demonstrating Stypoldione's proficient binding to its target. In summary, our study marks the inaugural comprehensive exploration of the potential molecular and pharmacological mechanisms underpinning Stypoldione's application in breast cancer treatment, employing a network pharmacology-based approach. These results imply that Stypoldione, due to its multifaceted impact on various targets and pathways, offers promise as an efficacious therapeutic choice for breast cancer.

## 7 Limitations

The absence of stypoldione as a commercially available substance was the study's only drawback. We were unable to conduct the in-vitro experiments since the chemical was not commercially available. We will investigate this medication more in the future to learn more about this molecule, though. Nevertheless, because this substance had showed promising outcomes in our research, the major goal of our study was to at least share our findings with the public.

## CRediT authorship contribution statement

**Hina Qayoom:** Data curation, Methodology, Project administration, Software, Validation, Visualization, Writing – original draft, Writing – review & editing. **Bader Alshehri:** Methodology, Project administration, Software, Validation, Visualization, Writing – review & editing. **Burhan Ul Haq:** Methodology, Project administration, Software, Validation, Visualization, Writing – review & editing. **Abdullah Almilaibary:** Project administration, Methodology, Software, Validation, Visualization, Writing – review & editing. **Mustfa Alkhanani:** Methodology, Project administration, Software, Validation, Visualization, Writing – review & editing. **Manzoor Ahmad Mir:** Conceptualization, Methodology, Project administration, Resources, Software, Supervision, Validation, Visualization, Writing – review & editing.

## Declaration of Competing Interest

The authors declare that they have no known competing financial interests or personal relationships that could have appeared to influence the work reported in this paper.
